# Organerhalt: Entscheidungskriterien für Patienten mit T3-Larynxkarzinom

**DOI:** 10.1007/s00106-022-01177-7

**Published:** 2022-05-16

**Authors:** Gerhard Dyckhoff, Rolf Warta, Christel Herold-Mende, Peter K. Plinkert, Heribert Ramroth

**Affiliations:** 1 Universitäts-Hals-Nasen-Ohrenklinik Heidelberg, Im Neuenheimer Feld 400, 69120 Heidelberg, Deutschland; 2 Neurochirurgische Universitätsklinik Heidelberg, Heidelberg, Deutschland; 3 Heidelberger Institut für Global Health, Universität Heidelberg, Heidelberg, Deutschland

**Keywords:** Laryngektomie, Kehlkopfkrebs, Alleinige Radiotherapie, Radiochemotherapie, Entscheidungsfindung, Laryngectomy, Laryngeal carcinoma, Radiotherapy alone, Chemoradiotherapy, Decision making

## Abstract

**Hintergrund:**

Derzeit gilt das größere, nicht mehr durch Teilresektionen behandelbare T3-Larynxkarzinom als optimaler Kandidat für einen Larynxorganerhalt (LP) mittels primärer Radiochemotherapie (pRCT). Wann wäre eine primäre Strahlentherapie (pRT) ggf. auch ohne Chemotherapiezusatz vertretbar, und wann selbst beim T3 doch eher die totale Laryngektomie mit risikoadaptierter adjuvanter Therapie (TL±aR[C]T) zu empfehlen?

**Methodik:**

In der Literatur wurde nach Parametern gesucht, die bei nichtrandomisierten zweiarmigen LP-Studien als Kriterium für den Einschluss in den chirurgischen anstelle des konservativen Arms genannt wurden oder die sich nach konservativer Therapie als signifikante Prognosemarker herausstellen. Hieraus Entwicklung eines Beratungsinstruments für die Therapieentscheidung.

**Ergebnisse:**

Als signifikante Prognosemarker beschrieben wurden das Tumorvolumen, das Vorhandensein und die Art der Stimmbandfixierung, das Ausmaß der Knorpelinfiltration, der N‑Status und die laryngeale Dysfunktion.

**Diskussion:**

Beim T3-Larynxkarzinom scheint eine reine pRT vertretbar zu sein, wenn das Volumen < 3,5 ccm für glottische bzw. < 6 ccm für supraglottische Tumoren beträgt und keine weiteren Risikofaktoren vorliegen. Die pCRT kann als Standard des konservativen LP bei Tumoren mit einem Volumen zwischen 6 ccm und 12 ccm gelten, bei einer Stimmbandfixierung Succo I/II, einer allenfalls minimalen Knorpelinfiltration und einer hohen nodulären Tumorlast. Bei Tumoren mit einem Volumen > 12 ccm, einem Stimmbandfixierungsmuster Succo III/IV, ausgedehnter oder multipler Knorpelinfiltration oder relevanter laryngealer Dysfunktion sollte primär die TL±a[C]RT empfohlen werden.

## Abstract (en)

**Background:**

By today’s standard, the optimal treatment of every individual tumor patient is discussed and determined in an interdisciplinary tumor board. According to the new S3 guidelines, larger volume T3 laryngeal cancers which are no longer safely resectable with larynx-sparing surgery are ideal candidates for a larynx preservation approach using primary chemoradiation (pCRT). So far, no clear criteria have been defined under what circumstances primary radiotherapy alone (pRT) might be acceptable in case chemotherapy (CT) is prohibited or in what cases, even in T3, upfront total laryngectomy with risk-adapted adjuvant treatment (TL±a[C]RT) should be recommended.

**Method:**

The literature was searched for parameters chosen as criteria for an inclusion in the surgical rather than the conservative arm in non-randomized LP studies or which proved to be significant prognostic markers after conservative treatment. Development of a counselling tool for therapeutic decision making.

**Results:**

Significant prognostic markers were tumor volume (< 3.5 ccm/< 6 ccm vs. 6–12 ccm vs. > 12 ccm), presence and kind of vocal cord fixation (none vs. Succo I/II vs. Succo III/IV), extent of cartilage infiltration (none vs. minimal vs. multiple/gross), nodal status (N0‑1 vs. N2-3), and laryngeal dysfunction (pretreatment necessity of feeding tube or tracheostomy).

**Conclusion:**

For T3 laryngeal cancers, pRT could be acceptable when the tumor volume is < 3.5 ccm for glottic and < 6 ccm for supraglottic tumors and there are no further risk factors. pCRT can be regarded as the standard for LP for tumors between 6 ccm and 12 ccm, vocal cord fixation Succo pattern I/II, only minimal cartilage infiltration and a high nodal burden. For tumor > 12 ccm, vocal cord fixation Succo pattern III/IV, gross or multiple cartilage infiltration or clinically relevant laryngeal dysfunction, upfront TL±a[C]RT should be considered.

Nach der neuen AWMF-S3-Leitlinie gilt das größere, nicht mehr durch Teilresektionen behandelbare T3-Larynxkarzinom als optimaler Kandidat für einen Larynxorganerhalt (LP) mittels primärer Radiochemotherapie (pRCT) [1]. In einer eigenen Kohortenstudie war für die Patienten mit T3-Larynxkarzinom kein signifikanter Überlebensunterschied zwischen primärer Radiochemotherapie (pRCT) und totaler Laryngektomie mit risikoadaptierter adjuvanter Therapie (TL±aR[C]T) nachweisbar, wohl aber im Vergleich zwischen alleiniger Bestrahlung (pRT) und (TL±aR[C]T); [2]. In unserem Kollektiv waren allerdings nur wenige T3-Patienten mit pRT behandelt worden, und es fand sich ein Selektionsbias hin zu älteren und krankeren Patienten, die beim Larynxorganerhalt (LP) ausschließlich durch pRT behandelt wurden. Für Patienten mit einer Kontraindikation für den Chemotherapiezusatz zur primären Bestrahlung stellt sich die Frage, ob und unter welchen Voraussetzungen möglicherweise dennoch eine reine Bestrahlung ohne relevanten Überlebensnachteil vertretbar sein könnte bzw. wann selbst beim T3-Karzinom eher die primäre TL erwogen werden sollte.

In der vorliegenden Arbeit wird ein Entscheidungsinstrument entwickelt, das bei dieser Fragestellung eine Hilfe sein könnte. Dabei besteht kein Anspruch auf Vollständigkeit der recherchierten Literatur, und eine klinische Prüfung der genannten Kriterien im Rahmen von klinischen, optimalerweise prospektiven Studien ist zu empfehlen.

## Methode

In 2018 stellten Tang et al. ein systematisches Review mit Metaanalyse von 11 Studien vor, die das Outcome von Patienten mit T3-Larynxkarzinom berichteten, die im direkten Vergleich entweder mittels TL operiert oder durch pRT bzw. pRCT behandelt worden waren. Diese 11 waren das Extrakt einer Datenbankabfrage von insgesamt 1298 Studien, die allein den zuvor definierten Einschlusskriterien entsprachen (im Einzelnen s. [3]). In diesen Studien und wiederum in der dort aufgeführten Literatur haben wir nach klinischen Parametern vonseiten des Tumors gesucht, die je nach Ausprägung für oder gegen den Einschluss in ein konservatives Organerhaltungsprogramm sprechen. Anschließend haben wir in einer PubMed-Recherche nach aktuelleren Publikationen zu den jeweiligen Kriterien gesucht und die Parameter entsprechend ergänzt bzw. korrigiert.

## Ergebnisse

Die im Folgenden besprochenen klinischen Parameter seitens des Tumors wurden als Entscheidungskriterium für oder gegen den Versuch eines LP-Ansatzes gefunden.

### Tumorvolumen

In mehreren Studien, die vergleichbare Ergebnisse zwischen LP und TL erreichen wollten, wurde für großvolumige Tumoren bewusst kein konservativer Therapieansatz empfohlen, z. B. [4, 5]. Al-Mamgani et al. beschrieben nach primär konservativer Therapie für größere T3-Tumoren eine Therapieversagerquote von 40 % (40/99), während sie für kleinere T3-Tumoren nur bei 6 % (4/71) lag [6]. Auffallend: Für Patienten nach pCRT lag die Versagerquote auch bei Tumoren mit größerem Volumen nur bei 11 %, nach alleiniger pRT dagegen bei 67 % (*p* < 0,001). Zwei unabhängige Studien ermittelten bei allerdings überwiegend supraglottischen Tumoren ein Volumen von 6 bzw. 8 ccm, ab dem die Radiatio zu signifikant schlechteren Ergebnissen führte [7, 8]. Für glottische Karzinome wurde hingegen nach alleiniger Radiatio eine signifikant bessere lokale Kontrolle mit Kehlkopferhalt bei einem deutlich geringeren Tumorvolumen von ≤ 3,5 ccm berichtet: (87 vs. 29 %; *p* = 0,0005; [9]). Alle 3 Studien sind recht alt (von 1997 bzw. 1999), erlauben aber eine Aussage über die untere Grenze des Tumorvolumens, bei der eine reine Radiatio noch vertretbar sein könnte. In neueren Studien wird das T3-Karzinom in der Regel mit RCT behandelt. Eine aktuelle Studie von Sharrett et al. beschreibt einen Cut-off von 12 ccm, ab dem auch die pCRT nicht mehr zu zufriedenstellenden Ergebnissen führt (lokoregionäres Therapieversagen nach nur 2 Jahren für Tumorvolumina > 12 ccm: 64 % vs. ≤ 12 ccm: 16 %, *p* = 0,006) [10]. Miyabe et al. bestimmten mittels ^18^F‑FDG-PET/CT das metabolisch aktive Tumorvolumen. Sie fanden einen deutlich höheren Cut-off von 28,7 ccm, ab dem das laryngektomiefreie Überleben (LFS) und das Gesamtüberleben (OS) nach pRCT signifikant schlechter war [11]. Allerdings waren in ihrem Kollektiv überwiegend Hypopharynxkarzinome und nur 41 % Larynxkarzinome enthalten, sodass sich für die hier besprochene Fragestellung keine verlässlichen Grenzen ableiten lassen. Allerdings korrelierte das Tumorvolumen besser mit dem Überleben als die T‑Kategorie (T3 oder T4). Daran angelehnt mag abweichend von dem definierten Einschlusskriterium (T3-Larynxkarzinom) auch die Studie von Hsin et al. herangezogen werden [12]. Sie untersuchten nur T4a-Larynxkarzinompatienten und fanden einen Cut-off für ein signifikant schlechteres Überleben bei 15 ccm, was recht gut mit dem Ergebnis von Sharrett et al. korreliert. Aus diesen Studien könnte man folgende Grenzen empfehlen, wobei aus Sicherheitsgründen die jeweils engere Grenze gewählt wird: Sollte der Chemotherapiezusatz für einen T3-Patienten kontraindiziert sein, so wäre bei Fehlen weiterer Risikofaktoren eine reine pRT zum Larynxorganerhalt bei kleinen Larynxkarzinomen beim supraglottischen Karzinom bis 6 ccm und beim glottischen bis 3,5 ccm vertretbar; zwischen 6 ccm und 12 ccm Tumorvolumen erscheint eine pCRT der geeignete Therapieansatz zu sein; für Tumoren > 12 cm sollte eher die primäre TL±a(C)RT erwogen werden. In der aktualisierten ASCO-Leitlinie von 2018 wird für „extensive T3 primary tumors“ die TL + aRT empfohlen, da hierdurch langfristig bessere Heilungsraten und auch bessere funktionelle Ergebnisse erreicht werden könnten [13].

### Stimmbandfixierung

Auch ein fixiertes Stimmband, das per se nach UICC (Union Internationale contre le Cancer) als T3-Kriterium gilt, wurde in einigen Studien als Kriterium angesehen, das eher gegen eine primär konservative Therapie sprach, z. B. [4, 5], insbesondere in Form einer ICRT [14]. Jedoch werden besonders nach CCRT auch günstige Ergebnisse berichtet: 65 % einer Serie von 23 Patienten erlangten wieder die komplette Stimmbandbeweglichkeit und hatten in diesem Fall danach ein 5‑Jahres-Gesamtüberleben von 100 % und ein 5‑Jahres-Überleben mit funktionserhaltenem Larynx von 87 % [15]. Allerdings gilt eine Stimmbandfixierung auch als stärkster Prädiktor für ein funktionell schlechtes Ergebnis nach pCRT: 56 % der Patienten mit Stimmbandfixierung benötigten 6 Monate nach Therapieabschluss noch eine Ernährungssonde und/oder ein Tracheostoma im Vergleich zu 6 % der Patienten ohne Fixierung [16]. Succo et al. beschreiben in ihrer 2019 veröffentlichten Arbeit 4 verschiedene Formen der Stellknorpelfixierung, die zum Stimmbandstillstand führen und die als Kriterium für eine Entscheidung zum konservativen Larynxorganerhalt genutzt werden können: War der Stellknorpel nur durch das Gewicht das darüber liegenden Tumors fixiert (Succo I) oder durch ein Befall des posterioren paraglottischen Raumes mit einer subglottischen Ausdehnung ≤ 10 mm (Succo II), so waren Stimmbandkarzinome trotz Stellknorpelfixierung erfolgreich mittels pCRT behandelbar. War dagegen die Articulatio cricoarytaenoidea betroffen und der Tumor reichte > 10 mm nach subglottisch (Succo III) oder war die Articulatio cricoarytaenoidea massiv involviert und der Tumor erreichte die Schleimhaut des Hypopharynx (Succo IV), so war der Tumor de facto wie ein T4-Karzinom anzusehen und die primäre TL wurde empfohlen [17]. Drei Jahre nach pCRT lag das Überleben mit laryngoösophagealer regelrechter Funktion bei 67 % bei Succo I, 80 % bei Succo II, 8 % bei Succo III und 0 % bei Succo IV (*p* < 0,001; [17]). Entsprechend könnte für Larynxkarzinome mit Stimmbandfixierung der Form Succo I und II die pCRT empfohlen werden, während im Falle einer Fixierung des Musters Succo III und IV die primäre TL nahegelegt werden sollte. Für eine reine pRT wäre eine Stimmbandfixierung hingegen als Kontraindikation anzusehen.

### Knorpelinfiltration

Larynxknorpel, der durch ein intaktes Perichondrium geschützt ist, hält auch hohe Strahlendosen aus. Eine Läsion der Knorpelhaut dagegen stellt ein hohes Risiko für eine Perichondritis und nachfolgende Chondronekrose dar [18]. Früher galt eine Knorpelinfiltration daher als relative Kontraindikation für eine Strahlentherapie. Nach Castelijns et al. bewirkte eine Larynxknorpelinfiltration mit Tumorvolumina von ≥ 5 ccm ein signifikant schlechteres Überleben (*p* < 0,05; [19]). Ab der 6. Auflage der UICC-Klassifizierung galt schon das „adjacent sign“ als T3-Kriterium, ein radiologisches Zeichen der unmittelbaren Nähe des Tumors zum Schildknorpel, das für eine Invasion des paraglottischen Raums mit oder ohne minimale Schildknorpelarrosion stand. Obschon bei Vorliegen dieses Zeichens noch nicht mal eine Knorpelinvasion als gesichert galt, stellte es sich als prognostisch hochgradiger Risikofaktor heraus, mit 5‑Jahres-Kontrollraten von 37 % mit „adjacent sign“ (daher als T3 klassifiziert) vs. 87 % ohne dieses Zeichen (daher T1-T2; *p* < 0,0001; [20]). Folglich wurde bei einem T3-Larynxkarzinom mit Knorpelinfiltration von einer alleinigen pRT grundsätzlich abgeraten und dieses als schlechte Indikation für eine pCRT angesehen [21]. Beim Konsenspanel für das Design klinischer Larynxorganerhalt-Studien wurde jedoch eine minimale Knorpelinfiltration nicht als Kontraindikation für die Teilnahme an einer LP-Studie gewertet [22]. Das Ausmaß der Knorpelinfiltration wird auch von Forastière et al. als entscheidendes Kriterium beschrieben: Ihre Beobachtung, dass die pCRT für T3-Patienten die geeignete Therapie sei, bezog sich explizit nicht auf Patienten mit ausgeprägter Knorpelinfiltration [23]. Die französische GETTEC-Studie ([14], nur T3, aber 100 % Stimmbandfixierung), wurde wegen des schlechten Ansprechens auf die pRCT abgebrochen: signifikant schlechteres Überleben als TL+aRT (*p* = 0,0006), 58 % TL im ICT-Arm vgl. 56 % TL der T4(!) im ICT-Arm in der VA-Studie. Die amerikanische VA-Studie (T1-T4 Tumoren, davon 65 % T3, aber < 60 % Stimmbandfixierung) dagegen wurde bei fehlendem Überlebensnachteil zu der grundlegenden LP-Studie überhaupt [24]. Beide Studien verwendeten die ICT gefolgt von pRT. Kamal et al. differenzierten zwischen keiner/geringer und multipler Knorpelinfiltration und beschrieb auch hier einen signifikanten Unterschied (5-Jahres-lokale-Kontrolle 95 vs. 72 %, *p* = 0,009; [25]). Gemäß diesen Studien könnte daher nur bei fehlender Knorpelinfiltration ggf. eine reine pRT als vertretbar angesehen werden; bei nur minimaler Knorpelinfiltration scheint eine pCRT möglich zu sein; bei ausgeprägter und vielfacher Knorpelinfiltration sollte dagegen die primäre TL erwogen werden. Dies entspricht der aktuellen Empfehlung der ASCO-Leitlinie: Bei „extensive laryngeal cartilage destruction“ ist auch beim T3 der TL der Vorzug zu geben [13].

### Laryngeale Dysfunktion

Unter laryngealer Dysfunktion wird nicht die Heiserkeit verstanden, die beim glottischen Karzinom ja schon als Frühsymptom auftritt. Nach dem o. g. Konsensuspapier von Lefebvre et al. galt als Ausschlusskriterium für einen LP-Ansatz eine bereits vor Therapiebeginn erforderliche Tracheotomie (Dyspnoe!) und/oder Ernährungssonde (Dysphagie!) oder rezidivierende, stationär zu behandelnde Pneumonien innerhalb der letzten 12 Monate (Aspirationsneigung!; [26]). Ebenso wäre auch die Aphonie zu nennen. Herchenhorn et al. aus Brasilien berichteten von einem signifikant schlechteren Überleben, wenn schon vor Beginn der pCRT eine Tracheotomie erforderlich war: 6 vs. 61 %; *p* = 0,001, mit einer für andere prognostische Faktoren adjustierten Hazard Ratio (HR) von 8,7 und einem 95 %-Konfidenzintervall (CI) von 3,1–24,9. Entsprechend wurde in diesem Falle eindringlich von dem Versuch eines Larynxorganerhalts abgeraten [27]. Was die Funktion anbelangt, so führt eine vor Therapiebeginn bereits erforderliche Tracheotomie auch dauerhaft zur Abhängigkeit von einem Tracheostoma (*p* < 0,0001) und ebenso von einer Ernährungssonde (*p* = 0,03; [28]). In derselben Studie war der onkologische Outcome jedoch nicht signifikant schlechter als bei Patienten ohne prätherapeutisches Tracheostoma [28]. Mendenhall et al. hingegen berichten nach Tracheotomie auch von einem signifikant schlechteren ursachenbezogenen Überleben (*p* = 0,0345; [9]). In der aktualisierten ASCO-Leitlinie wird empfohlen, bei prätherapeutischer schwerer oder wiederholter Aspiration, funktionsuntüchtiger Stimme oder behindertem Luftweg sowie schwerer Kehlkopfdysfunktion auch beim T3 die TL vorzuziehen, weil dadurch besser onkologische und funktionelle Ergebnisse erzielt werden könnten [13].

### N-Status

Der Lymphknotenstatus ist kein direktes Kriterium für oder gegen einen konservativen Larynxorganerhalt. Der N‑Status ist aber ein entscheidender Parameter, wenn es um die Frage geht, ob die pRT nur mit oder auch ohne Chemotherapie empfohlen werden kann. In vielen Studien wird der N‑Status (insbesondere N2-N3) als unabhängiger negativer prognostischer Faktor beschrieben, z. B. [4, 5, 29, 30]. In unserer Kohortenstudie fand sich für ein fortgeschrittenes N‑Stadium eine HR von 3,5 (95%-KI 2,2–5,7; [31]). Nach dem niederländischen Konsensusprotokoll erhalten Patienten mit N2-3-Status grundsätzlich statt einer pRT eine pCRT [32]. Dadurch wurde für T3-Patienten ein praktisch identisches Outcome für T3N0‑1 nach pRT wie für T3N2‑3 nach pCRT erreicht (5-Jahres-Gesamtüberleben von 45 vs. 47 %, *p* = 0,54; [33]). Alternativ wäre bei einem Lymphknotenstatus N2‑3 aber Kontraindikation für Cisplatin eine Kombination aus primärer Neck dissection und anschließender pRT des Primärtumors mit aRT der Lymphabflusswege denkbar.

Empfehlungen, die sich aus den genannten Studien für die Therapieentscheidung LP vs. TL±a(C)RT ergeben, sind zusammengefasst in Tab. 1.

**Table 1:** 

Parameter	*Niedriges Risiko* (Bei KI für Chemo *pRT alleine* möglicherweise vertretbar)	*Mittleres Risiko* (*pCRT*, bei KI für Chemo TL erwägen!)	*Hohes Risiko* (*TL* erwägen!)
Tumorvolumen [4–10]	< 3,5 ccm (glottisch) < 6 ccm (supraglottisch)	6 ccm ≤ x ≤ 12 ccm	> 12 ccm
Stimmbandfixierung [4, 5, 14–17, 34]	Keine	Fixierungsmuster Succo I + II	Fixierungsmuster Succo III + IV
Knorpelinfiltration [13, 14, 19–23, 25, 26, 35]	Keine	Minimal	Multiple/ausgedehnte
Laryngeale Dysfunktion [9, 13, 26–28]	n.a.	n.a.	Ernährungssonde, Tracheostoma, wiederholte Pneumonien in den letzten 12 Monaten
N‑Stadium [4, 5, 29–31, 33, 36]	N0‑1	N2‑3	n.a.

*n.a.* nicht anwendbar, *KI* Kontraindikation, *pRT* primäre Radiotherapie, *pCRT* primäre Radiochemotherapie, *TL* totale Laryngektomie, *N* nodus lymphaticus, Lymphknotenmetastasierung, *Succo* Muster der Stimmbandfixierung gemäß [17]

## Diskussion

Bei der kritischen Durchsicht der von Tang et al. genannten 11 Studien [3] fielen 2 unterschiedliche Studientypen auf: Typ 1 hat das erklärte Ziel, beim konservativen Larynxorganerhalt vergleichbar gute Ergebnisse zu erlangen wie durch eine TL. Hierbei wird implizit vorausgesetzt, dass die Effektivität der beiden Ansätze unterschiedlich sei. Betont wurde hier die richtige Patientenauswahl für den Larynxorganerhalt, typische Beispiele hierfür sind [4, 5, 36]. Inwieweit aus Studien diesen Typs in einer Metaanalyse die Effektivität der beiden Therapieverfahren sinnvoll verglichen werden kann, ist allerdings eher fraglich. Studientyp 2 zielt auf den echten Vergleich der Effektivität der beiden Therapiestrategien. In den genannten Arbeiten waren dies allerdings sämtlich retrospektive Kohortenstudien, z. B. [37–39], bei denen ein Einschlussbias in die verschiedenen Studienarme nicht ausgeschlossen werden kann. In Reinform wären Typ-2-Studien prospektive, randomisierte klinische Studien wie die grundlegenden Larynxorganerhaltstudien [23, 24]; hier wurden jedoch die Tumorkategorien T2-T4 gemeinsam ausgewertet, sodass eine Aussage zu der spezifischen Kategorie T3 wie von Tang et al. vorausgesetzt nicht möglich ist. Klinische Kriterien von Seiten des Patienten (Alter, Allgemeinzustand, Komorbiditäten, spezifische Laborparameter, etc.) waren nicht Teil dieser Arbeit.

Die Kriterien für die Primärtumorklassifikation des Larynx haben sich im Laufe der verschiedenen Auflagen der TNM-Klassifikation (UICC wie auch AJCC) gewandelt. Dies gilt insbesondere für die Schildknorpelinfiltration. Bis 2002 war diese ein T4-Kriterium, danach aber nur noch eines für einen T3-Tumor; erst der Knorpeldurchbruch führt zur T4-Klassifikation. Da viele Studien über lange Zeiträume rekrutierten, während derer sich die Klassifikation änderte, musste zur korrekten Auswertung eine Reklassifizierung der T3/T4 erfolgen (vgl. [36]). Kritisch ist auch die c‑Klassifikation aufgrund der Bildgebung, die ja im Falle der konservativen LP-Therapie nicht pathologisch verifiziert wird. Hierdurch kann es tendenziell zu einem Overstaging bei der konservativen Therapie kommen, insbesondere wenn schon bei „Verdacht auf“ Knorpelinfiltration in der Bildgebung ein T3 angenommen wird.

Die in dieser Arbeit genannte pRCT für den Organerhalt in den unterschiedlichen Studien ist kein einheitlicher Therapieansatz. In der S3-Leitlinie werden verschiedene Strahlentherapieschemata und ebenso unterschiedliche Chemotherapeutika zur Kombination genannt, z. B. Cisplatin, Carboplatin, Mitomycin, 5‑Fluorouracil und Docetaxel, die entweder synchron oder als Induktion eingesetzt werden können [1]. Als Standardchemotherapeutikum zumindest für die simultane pRCT gilt Cisplatin.

Ob bei einem Patienten mit Chemotherapieunfähigkeit, z. B. bei Nierenfunktionsstörung, auch bei mittlerem Risiko nicht doch ein Larynxorganerhalt durchgeführt werden kann, hängt entscheidend davon ab, ob es eine zumindest annähernd gleichwertige Alternative für Cisplatin gibt. In erster Linie wird hier Cetuximab diskutiert. Der monoklonale Antikörper gegen den epidermalen Wachstumsfaktor zeigt zwar ein anderes Nebenwirkungsprofil als die Platinverbindung (Cetuximab: dermatotoxisch, i.S. eines akneiformen Hautausschlags; Cisplatin: hämatotoxisch, nephrotoxisch und gastrointestinal), sodass es auch bei eingeschränkter Nierenfunktion eingesetzt werden kann. In der S3-Leitlinie wird es als „beim Larynxkarzinom nicht vorrangig einzusetzen“ bezeichnet, da der Effekt in der Studie von Bonner et al. „relativ klein“ gewesen sei. Die Radioimmuntherapie (RIT) mit Cetuximab bewirkte in der Bonner-Studie beim Oropharynxkarzinom ein signifikant besseres Überleben als die alleinige pRT: Das mediane Überleben beim Oropharynxkarzinom lag bei > 66,0 vs. 30,3 Monaten (HR 0,62), beim Larynxkarzinom fand sich dagegen kein relevanter Unterschied (32,8 vs. 31,6 Monate; HR 0,87; [40]). Dasselbe gilt für den Larynxerhalt nach 2 Jahren: Mit 87,9 vs. 85,7 % war der Unterschied klinisch nicht relevant (HR 0,57; 95 %-KI 0,23–1,42; *p* = 0,22). Die Toxizität von Cetuximab hingegen kann erheblich sein und führte in einer italienischen Studie sogar zu mehr Studienabbrüchen und mehr Todesfällen als Cisplatin [41]. Auch im Langzeitverlauf führte die RIT im Vergleich zur alleinigen pRT zu keinem signifikanten Unterschied für den Larynxorganerhalt oder das laryngektomiefreie Überleben [42]. In der TREMPLIN-Studie (RIT mit Cetuximab vs. RCT mit Cisplain, beides nach Induktionschemotherapie [IC]) zeigte sich im RIT-Arm eine höhere Lokalrezidivrate als bei der RCT, und das Gesamtansprechen beider war nicht besser als für pRT alleine nach IC [43–45]. Insgesamt stellt Cetuximab daher eher keinen geeigneten Ersatz für Cisplatin dar.

Die englische Leitlinie sieht zum Organerhalt beim T3-Larynxkarzinom grundsätzlich die pCRT vor [46]. Im Falle einer Kontraindikation für den Chemotherapiezusatz aufgrund multipler Komorbiditäten, insbesondere einer eingeschränkten Nierenfunktion und reduzierten Allgemeinzustandes oder fortgeschrittenen Alters, wird die TL ± aRT empfohlen. Lin et al. fanden im Nordosten Englands beim T3-Larynxkarzinom ein signifikant schlechteres Überleben nach alleiniger pRT im Vergleich zur pCRT. Aufgrund dieser Erfahrung empfahlen die Autoren den Patienten die konkreten Überlebenszahlen vor Augen zu führen, um sie in der Entscheidung für die primäre TL zu motivieren: die 5‑Jahres-Überlebensrate nach alleiniger pRT lag bei 13 % im Vergleich zu 48 % nach pCRT [47]. Allerdings kumulieren ohne Randomisierung bei den konservativ behandelten Patienten (insbesondere denen, die ohne Chemotherapie behandelt wurden) alte und multimorbide Patienten und nichtresektable Tumoren. Dieser Bias ist auch in unserer eigenen Kohortenstudie anzunehmen [2].

Es besteht kein Anspruch auf Vollständigkeit der recherchierten Literatur. Daher können die erarbeiteten Kriterien zur Therapieentscheidung nur mit Vorbehalt zu einer Behandlungsempfehlung führen. Sie stellen aber eine gute Grundlage zur „hypothesis generation“ für künftige prospektive Studien dar, in denen die vorgeschlagenen Auswahlkriterien validiert werden sollten.

## Fazit für die Praxis

- Bei Kontraindikation für eine Chemotherapie kann bei klinisch weniger fortgeschrittenen T3-Karzinomen nach den angeführten Studien eine *primäre alleinige RT* unter folgenden Voraussetzungen vertretbar sein: Volumen < 3,5 ccm für glottische und < 6 ccm für supraglottische Tumoren, keine Stimmbandfixierung, keine Knorpelinfiltration und als Lymphknotenstatus N0‑1.
- Größere Tumoren bis 12 ccm, mit Stimmbandfixierung nach Succo I oder II und nur minimaler Knorpelinfiltration sind eine gute Indikation für eine *pCRT*, bei einer Kontraindikation für Chemotherapie hingegen ist eher die TL±aRT zu erwägen.
- Bei Tumoren > 12 ccm, Stimmbandfixierung nach Succo III oder IV, multipler oder ausgedehnter Knorpelinfiltration und Notwendigkeit der Tracheotomie oder Sondenernährung bereits vor Therapiebeginn wäre die primäre* TL+aR(C)T* zu empfehlen.
- Cisplatin gilt beim Larynxkarzinom generell als Chemotherapeutikum der Wahl für die Kombination mit der pRT, Cetuximab scheint keine gleichwertige Alternative für Cisplatin zu sein.

## References

[1] Bootz F (2019) S3-Leitlinie Diagnostik, Therapie und Nachsorge des Larynxkarzinoms. https://www.awmf.org/uploads/tx_szleitlinien/017-076OLl_S3_Larynxkarzinom_2019-11.pdf. Zugegriffen: 15.12.2021. (Leitlinienprogramm Onkologie. AWMF online)

[2] Dyckhoff G, Warta R, Herold-Mende C, Winkler V, Plinkert PK, Ramroth H (2021). Chemoradiotherapy but not radiotherapy alone for larynx preservation in T3. Considerations from a German observational cohort study. Cancers (Basel).

[3] Tang ZX, Gong JL, Wang YH, Li ZH, He Y, Liu YX, Zhou XH (2018). Efficacy comparison between primary total laryngectomy and nonsurgical organ-preservation strategies in treatment of advanced stage laryngeal cancer: a meta-analysis. Medicine.

[4] Nguyen-Tan PF, Le QT, Quivey JM, Singer M, Terris DJ, Goffinet DR, Fu KK (2001). Treatment results and prognostic factors of advanced T3-4 laryngeal carcinoma: the university of California, San Francisco (UCSF) and Stanford university hospital (SUH) experience. Int J Radiat Oncol Biol Phys.

[5] Timme DW, Jonnalagadda S, Patel R, Rao K, Robbins KT (2015). Treatment selection for T3/T4a laryngeal cancer: chemoradiation versus primary surgery. Ann Otol Rhinol Laryngol.

[6] Al-Mamgani A, Tans L, van Rooij P, Levendag PC (2012). A single-institutional experience of 15 years of treating T3 laryngeal cancer with primary radiotherapy, with or without chemotherapy. Int J Radiat Oncol Biol Phys.

[7] Mancuso AA, Mukherji SK, Schmalfuss I, Mendenhall W, Parsons J, Pameijer F, Hermans R, Kubilis P (1999). Preradiotherapy computed tomography as a predictor of local control in supraglottic carcinoma. J Clin Oncol.

[8] Hermans R, Van den Bogaert W, Rijnders A, Baert AL (1999). Value of computed tomography as outcome predictor of supraglottic squamous cell carcinoma treated by definitive radiation therapy. Int J Radiat Oncol Biol Phys.

[9] Mendenhall WM, Parsons JT, Mancuso AA, Pameijer FJ, Stringer SP, Cassisi NJ (1997). Definitive radiotherapy for T3 squamous cell carcinoma of the glottic larynx. J Clin Oncol.

[10] Sharrett JM, Ward MC, Murray E, Scharpf J, Lamarre ED, Prendes BL, Lorenz RR, Burkey BB, Koyfman SA, Woody NM (2020). Tumor volume useful beyond classic criteria in selecting larynx cancers for preservation therapy. Laryngoscope.

[11] Miyabe J, Hanamoto A, Tatsumi M, Hamasaki T, Takenaka Y, Nakahara S, Kishikawa T, Suzuki M, Takemoto N, Michiba T (2017). Metabolic tumor volume of primary tumor predicts survival better than T classification in the larynx preservation approach. Cancer Sci.

[12] Hsin LJ, Fang TJ, Tsang NM, Chin SC, Yen TC, Li HY, Liao CT, Chen IH (2014). Tumor volumetry as a prognostic factor in the management of T4a laryngeal cancer. Laryngoscope.

[13] Forastiere AA, Ismaila N, Wolf GT (2018). Use of larynx-preservation strategies in the treatment of laryngeal cancer: American society of clinical oncology clinical practice guideline update summary. J Oncol Pract.

[14] Richard JM, Sancho-Garnier H, Pessey JJ, Luboinski B, Lefebvre JL, Dehesdin D, Stromboni-Luboinski M, Hill C (1998). Randomized trial of induction chemotherapy in larynx carcinoma. Oral Oncol.

[15] Solares CA, Wood B, Rodriguez CP, Lorenz RR, Scharpf J, Saxton J, Rybicki LA, Strome M, Esclamado R, Lavertu P (2009). Does vocal cord fixation preclude nonsurgical management of laryngeal cancer?. Laryngoscope.

[16] Staton J, Robbins KT, Newman L, Samant S, Sebelik M, Vieira F (2002). Factors predictive of poor functional outcome after chemoradiation for advanced laryngeal cancer. Otolaryngol Head Neck Surg.

[17] Succo G, Cirillo S, Bertotto I, Maldi E, Balmativola D, Petracchini M, Gned D, Fornari A, Motatto GM, Sprio AE (2019). Arytenoid fixation in laryngeal cancer: radiological pictures and clinical correlations with respect to conservative treatments. Cancers (Basel).

[18] Becker M (2000). Neoplastic invasion of laryngeal cartilage: radiologic diagnosis and therapeutic implications. Eur J Radiol.

[19] Castelijns JA, Becker M, Hermans R (1996). Impact of cartilage invasion on treatment and prognosis of laryngeal cancer. Eur Radiol.

[20] Murakami R, Nishimura R, Baba Y, Furusawa M, Ogata N, Yumoto E, Yamashita Y (2005). Prognostic factors of glottic carcinomas treated with radiation therapy: value of the adjacent sign on radiological examinations in the sixth edition of the UICC TNM staging system. Int J Radiat Oncol Biol Phys.

[21] Gomez Serrano M, Iglesias Moreno MC, Gimeno Hernandez J, Ortega Medina L, Martin Villares C, Poch Broto J (2016). Cartilage invasion patterns in laryngeal cancer. Eur Arch Otorhinolaryngol.

[22] Lefebvre JL, Ang KK (2009). Larynx preservation clinical trial design: key issues and recommendations—a consensus panel summary. Head Neck.

[23] Forastiere AA, Goepfert H, Maor M, Pajak TF, Weber R, Morrison W, Glisson B, Trotti A, Ridge JA, Chao C (2003). Concurrent chemotherapy and radiotherapy for organ preservation in advanced laryngeal cancer. N Engl J Med.

[24] Wolf G (1991). Induction chemotherapy plus radiation compared with surgery plus radiation in patients with advanced laryngeal cancer. N Engl J Med.

[25] Kamal M, Ng SP, Eraj SA, Rock CD, Pham B, Messer JA, Garden AS, Morrison WH, Phan J, Frank SJ (2018). Three-dimensional imaging assessment of anatomic invasion and volumetric considerations for chemo/radiotherapy-based laryngeal preservation in T3 larynx cancer. Oral Oncol.

[26] Lefebvre JL, Ang KK, Larynx Preservation Consensus Panel (2009). Larynx preservation clinical trial design: key issues and recommendations—a consensus panel summary. Head Neck.

[27] Herchenhorn D, Dias FL, Ferreira CG, Araujo CM, Lima RA, Small IA, Kligerman J (2008). Impact of previous tracheotomy as a prognostic factor in patients with locally advanced squamous cell carcinoma of the larynx submitted to concomitant chemotherapy and radiation. ORL J Otorhinolaryngol Relat Spec.

[28] Jefferson GD, Wenig BL, Spiotto MT (2016). Predictors and outcomes for chronic tracheostomy after chemoradiation for advanced laryngohypopharyngeal cancer. Laryngoscope.

[29] Hanna J, Brauer PR, Morse E, Mehra S (2019). Margins in laryngeal squamous cell carcinoma treated with transoral laser microsurgery: a national database study. Otolaryngol Head Neck Surg.

[30] Patel SA, Qureshi MM, Dyer MA, Jalisi S, Grillone G, Truong MT (2019). Comparing surgical and nonsurgical larynx-preserving treatments with total laryngectomy for locally advanced laryngeal cancer. Cancer.

[31] Ramroth H, Schoeps A, Rudolph E, Dyckhoff G, Plinkert P, Lippert B, Feist K, Delank KW, Scheuermann K, Baier G (2011). Factors predicting survival after diagnosis of laryngeal cancer. Oral Oncol.

[32] Kaanders JH, Hordijk GJ, Dutch Cooperative Head and Neck Oncology Group (2002). Carcinoma of the larynx: the Dutch national guideline for diagnostics, treatment, supportive care and rehabilitation. Radiother Oncol.

[33] Timmermans AJ, van Dijk BA, Overbeek LI, van Velthuysen ML, van Tinteren H, Hilgers FJ, van den Brekel MW (2016). Trends in treatment and survival for advanced laryngeal cancer: a 20-year population-based study in the Netherlands. Head Neck.

[34] Porter MJ, McIvor NP, Morton RP, Hindley AC (1998). Audit in the management of T3 fixed-cord laryngeal cancer. Am J Otolaryngol.

[35] Castelijns JA, van den Brekel MW, Tobi H, Smit EM, Golding RP, van Schaik C, Snow GB (1996). Laryngeal carcinoma after radiation therapy: correlation of abnormal MR imaging signal patterns in laryngeal cartilage with the risk of recurrence. Radiology.

[36] Timmermans AJ, de Gooijer CJ, Hamming-Vrieze O, Hilgers FJ, van den Brekel MW (2015). T3-T4 laryngeal cancer in the Netherlands cancer institute; 10-year results of the consistent application of an organ-preserving/-sacrificing protocol. Head Neck.

[37] Patel UA, Howell LK (2011). Local response to chemoradiation in T4 larynx cancer with cartilage invasion. Laryngoscope.

[38] Dziegielewski PT, O’Connell DA, Klein M, Fung C, Singh P, Alex Mlynarek M, Fung D, Harris JR, Seikaly H (2012). Primary total laryngectomy versus organ preservation for T3/T4a laryngeal cancer: a population-based analysis of survival. J. Otolaryngol. Head Neck Surg..

[39] Bussu F, Paludetti G, Almadori G, De Virgilio A, Galli J, Micciche F, Tombolini M, Rizzo D, Gallo A, Giglia V (2013). Comparison of total laryngectomy with surgical (cricohyoidopexy) and nonsurgical organ-preservation modalities in advanced laryngeal squamous cell carcinomas: a multicenter retrospective analysis. Head Neck.

[40] Bonner JA, Harari PM, Giralt J, Azarnia N, Shin DM, Cohen RB, Jones CU, Sur R, Raben D, Jassem J (2006). Radiotherapy plus cetuximab for squamous-cell carcinoma of the head and neck. N Engl J Med.

[41] Magrini SM, Buglione M, Corvo R, Pirtoli L, Paiar F, Ponticelli P, Petrucci A, Bacigalupo A, Crociani M, Lastrucci L (2016). Cetuximab and radiotherapy versus cisplatin and radiotherapy for locally advanced head and neck cancer: a randomized phase II trial. J Clin Oncol.

[42] Bonner JA, Harari PM, Giralt J, Cohen RB, Jones CU, Sur RK, Raben D, Baselga J, Spencer SA, Zhu J (2010). Radiotherapy plus cetuximab for locoregionally advanced head and neck cancer: 5-year survival data from a phase 3 randomised trial, and relation between cetuximab-induced rash and survival. Lancet Oncol.

[43] Lefebvre JL, Pointreau Y, Rolland F, Alfonsi M, Baudoux A, Sire C, de Raucourt D, Malard O, Degardin M, Tuchais C (2013). Induction chemotherapy followed by either chemoradiotherapy or bioradiotherapy for larynx preservation: the TREMPLIN randomized phase II study. J Clin Oncol.

[44] Bonomi MR, Blakaj A, Blakaj D (2018). Organ preservation for advanced larynx cancer: a review of chemotherapy and radiation combination strategies. Oral Oncol.

[45] Pointreau Y, Garaud P, Chapet S, Sire C, Tuchais C, Tortochaux J, Faivre S, Guerrif S, Alfonsi M, Calais G (2009). Randomized trial of induction chemotherapy with cisplatin and 5-fluorouracil with or without docetaxel for larynx preservation. J Natl Cancer Inst.

[46] Jones TM, De M, Foran B, Harrington K, Mortimore S (2016). Laryngeal cancer: United Kingdom national multidisciplinary guidelines. J Laryngol Otol.

[47] Lin DJ, Goodfellow M, Ong J, Chin MY, Lazarova L, Cocks HC (2020). Treatment outcomes of laryngectomy compared to non-surgical management of T3 laryngeal carcinomas: a 10-year multicentre audit of 179 patients in the northeast of England. J Laryngol Otol.

